# A novel HPV mRNA vaccine candidate: immunogenicity and definition of immune correlates of protection

**DOI:** 10.3389/fimmu.2026.1925901

**Published:** 2026-10-21

**Authors:** Hyogyeong Hwang, Timothy An, Eun-Hye Seo, Pil-Gu Park, Kee-Jong Hong

**Affiliations:** 1 Department of Bio-Medical Science, GAIST, Gachon University, Incheon, Republic of Korea; 2 Department of Microbiology, College of Medicine, Gachon University, Incheon, Republic of Korea; 3 Department of Life Science, College of BioNano Technology, Gachon University, Seongnam, Republic of Korea; 4 Lee Gil Ya Cancer and Diabetes Institute, Gachon University, Incheon, Republic of Korea

**Keywords:** human papillomavirus, immune correlates of protection, mRNA vaccine, prophylactic vaccine, standardized assay

## Abstract

Human papillomavirus (HPV), the major cause of cervical cancer, continues to be associated with an increasing number of diagnosed cases despite the availability of licensed vaccines such as Cervarix™ and GARDASIL^®^. In addition, cervical cancer incidence remains especially high in low- and middle-income countries, while vaccine coverage remains low due to high costs and instability in supply. We developed a novel HPV vaccine candidate using mRNA platform and to propose immune correlates of protection (CoP) for efficacy evaluation. A protection threshold was established based on GARDASIL^®^ 9, and the corresponding antibody titers were determined. These antibody titers were used as Reference for the immunogenicity analysis of the generated HPV16 mRNA. As a result, the vaccine was shown to induce a sufficiently significant level of protective efficacy. Furthermore, based on the immunogenicity data obtained from GARDASIL^®^ 9 and HPV16 mRNA, the minimum antibody titers required for protection were determined, thereby defining CoP for HPV vaccine. Additionally, HPV16 mRNA induced cellular immune responses associated with CD4- and CD8-specific cytokine secretion, suggesting potential protective mechanism in which cellular immunity complements humoral immunity. This study proposes an objective evaluation framework for comparing and analyzing HPV vaccine efficacy across different vaccine platforms.

## 1 Introduction

Human Papillomavirus (HPV) is a non-enveloped virus with over 100 subtypes (1). It is primarily transmitted through sexual contact and can cause benign or malignant lesions (2). Persistent infection with HPV is the leading cause of cervical cancer in women, accounting for over 90% of cases (1, 3, 4). The major capsid protein L1 mediates HPV attachment to and entry into host cells and serves as the primary target of prophylactic vaccines (5, 6). Commercially available HPV vaccines, such as Cervarix™ and GARDASIL^®^, are recombinant protein vaccines using L1 virus-like particles (VLPs) (3, 7). However, their high cost restricts vaccine uptake in low- and middle-income countries as well as in Korea, despite the rising incidence of cervical cancer (8–11). Therefore, the development of new platform-based HPV vaccines capable of overcoming the limitations of existing vaccines is required.

mRNA vaccines began to attract significant attention during COVID-19 pandemic as their efficacy and efficiency were demonstrated (12). This platform can be rapidly designed and manufactured at large scale, which may enable them to be cost-effective (13). In addition, mRNA vaccines also have the advantage of enhancing vaccine efficacy through the intrinsic immunostimulatory effects of nucleic acids, without the need for additional adjuvants (14, 15). HPV VLPs exhibit type-specific surface structural characteristics, and accordingly, the optimal conditions required to efficiently induce self-assembly may vary among HPV types (16, 17). Therefore, individual process development and optimization may be required for each HPV type. These characteristics can lead to increased manufacturing costs as well as prolonged development and production timelines. In contrast, once mRNA vaccine platform has been established, new vaccines can be readily designed by simply replacing the genetic sequence encoding the target HPV type, thereby offering temporal and economic advantages over VLP-based vaccines in terms of development and manufacturing. Based on the advantages of mRNA vaccines, we established an mRNA-based platform as a novel HPV vaccine candidate to overcome the limitations of existing HPV vaccines.

Currently, the efficacy of HPV vaccines is primarily evaluated based on the incidence of cervical precancerous lesions, including cervical intraepithelial neoplasia grades 2 and 3 (CIN2/3) and adenocarcinoma *in situ* (AIS) (18). However, progression from HPV infection to cervical cancer typically requires several years, and more than 90% of HPV infections resolve spontaneously within two years (18–20). Consequently, efficacy assessment based on disease incidence necessitates long-term follow-up, leading to prolonged study durations as well as increased costs and logistical complexity. Moreover, because HPV infection also causes genital diseases in men, disease-based endpoints focused on cervical lesions may not fully capture the overall protective efficacy of HPV vaccines (21). Accordingly, using GARDASIL^®^ 9 as Reference, we defined the minimum antibody titer required to inhibit viral infection and established antibody quantification methods based on ELISA and neutralization assay.

In HPV infection, as protective antibodies are known to recognize conformational epitopes formed by the capsomere structure composed of L1 pentamers, virus-like particles (VLPs) with intact conformational structures were used as antigens in ELISA (22, 23). Neutralizing antibody responses were assessed using luciferase-based pseudovirus neutralization assay (PBNA) to ensure robustness and experimental convenience (24, 25). To establish a standardized approach for evaluating the efficacy of HPV vaccine candidates, these assays were standardized to quantitatively assess vaccine-induced binding and neutralizing antibody responses. By relating these antibody responses to protection *in vivo* HPV PsV challenge model, antibody-based immune correlates of protection (CoP) were established to provide quantitative criteria for evaluation of HPV vaccine efficacy.

In this study, we established standardized assays and CoP for the evaluation of HPV vaccine efficacy and applied these to a novel HPV mRNA vaccine candidate to assess its immunogenicity. In addition to antibody responses, based on previous studies demonstrating that mRNA vaccines elicit both humoral and cellular immune responses, T cell–mediated immunity was additionally evaluated (12, 13). Notably, mRNA vaccines have been reported to preferentially induce Th1-biased immune responses (26, 27). To evaluate these immunological characteristics, antigen-specific IgG subclass analysis was performed. Collectively, this study supports the reliability of the proposed CoP and analytical assays and provide a quantitative framework for the objective assessment of HPV vaccine candidates, including mRNA-based platforms.

## 2 Materials and methods

### 2.1 Preparation and characterization of mRNA vaccine

HPV16 L1 sequence (accession number: P03101) from p16sheLL plasmid, which is commonly used for PsV production, was used as a template in this study (28). CUK3–1 plasmid vector was provided by Catholic University (Seoul, Korea). The target coding region was inserted into CUK3–1 plasmid vector. This vector included T7 promoter, 5’- and 3’-untranslated regions (UTRs), and a 100-nucleotide poly-A tail necessary for *in vitro* transcription (IVT). To improve mRNA stability and translational efficiency, a 20-nucleotides linker element (A50L50LO) was incorporated within the poly-A tail (29). The plasmids were linearized by restriction enzyme digestion for 2 hours at 37 °C and purified using QIAquick PCR & Gel clean up kit (QIAGEN, Hilden, Germany) prior to IVT. mRNA was synthesized using HiScribe^®^ T7 High Yield RNA Synthesis Kit (NEB, Ipswich, USA) with CleanCap (TriLink, San Diego, USA) to enhance stability. Synthesized mRNA was purified by lithium chloride (LiCl) precipitation and treated with double-stranded RNA removal kit (CELLSCRIPT, Madison, USA). mRNA concentration was measured using NanoDrop2000 v1.5 (Thermofisher, Waltham, USA).

### 2.2 Denaturing agarose gel electrophoresis

mRNA samples were mixed with RNA loading dye and heated at 70 °C for 10 minutes. RNA (100 ng per lane) and ladder were loaded onto a 1.2% agarose gel containing 2.2 M formaldehyde in 1X MOPS-EDTA-Sodium Acetate (MESA) buffer (Sigma-Aldrich, St. Louis, USA) and electrophoresed at 100 V for approximately 35 minutes. The images were acquired using Gel Doc (Bio-Rad, Hercules, USA) and analyzed with Image Lab v5.2.

### 2.3 Dot blot

500 ng of RNA was spotted onto positively charged nylon membrane (Sigma-Aldrich, St. Louis, USA) and allowed to air-dry. The membrane was blocked with 5% skim milk in TBST for 1 hour, followed by incubation with J2 anti-dsRNA antibody (Cell Signaling Technology, Danvers, USA) (1:1,000 in 1% skim milk in TBST) for 1 hour. After three washing with TBST, the membrane was incubated with HRP-conjugated anti-mouse IgG secondary antibody (1:3,000 in 1% skim milk in TBST) for 1 hour. Signals were detected using ECL substrate and quantified using Image J v1.54g.

### 2.4 Cell transfection

HEK293TT cells, provided by Yonsei University (Seoul, Korea) were cultured in DMEM with 10% FBS, 1% NEAA, 1% Glutamax, and 1% antibiotic-antimycotic. Cells at 70–80% confluency were transfected with 4 μg of mRNA using Lipofectamine 2000 (Invitrogen, Waltham, USA). 24 hours post-transfection, cells were lysed with RIPA buffer containing protease inhibitors. Protein concentration was quantified via BCA assay.

### 2.5 Western blot

Proteins (10 μg) were mixed with loading buffer and denatured at 95 °C for 5 minutes. Denatured samples were separated by 10% SDS-PAGE and transferred to nitrocellulose membranes using iBlot2 (Invitrogen, Waltham, USA). Membranes were blocked with 5% skim milk in TBST for 1 hour at room temperature. HPV pan-specific primary antibodies (Invitrogen, Waltham, USA) were diluted 1:1,000 in 5% BSA, 1% sodium azide in TBST and incubated overnight at 4 °C. HRP-conjugated secondary antibodies were diluted 1:10,000 in 5% skim milk and incubated 1 hour at room temperature. Protein bands were visualized with ECL substrate by Amersham™ ImageQuant™ 800 (Cytiva, Marlborough, USA).

### 2.6 Lipid nanoparticle encapsulation of mRNA vaccine

LNP encapsulation was performed at Seoul National University Hospital (Seoul, Korea). Lipid composition included SM-102 (ionizable lipids) (MedChemExpress, Monmouth Junction USA), DSPC (1,2-distearoyl-sn-glycero-3-phosphocholine), cholesterol and DMG-PEG2000 (1,2-dimyristoyl-rac-glycero-3-methoxypolyethylene glycol-2000) at a molar ratio of 50:10:38.5:1.5. mRNA was mixed with lipids in 50 mM sodium citrate buffer (pH 4.0), and LNPs were generated using enCELL-Master V2 (ENPARTICLE, Busan, Korea) under a 3:1 aqueous-to-organic flow ratio at a total flow rate of 10 mL/min. The resulting LNPs were subsequently concentrated and transferred into PBS using Amicon Ultra centrifugal filter units (Merck, Darmstadt, Germany). For quality control, the particle size, polydispersity index (PDI), zeta potential, and encapsulation efficiency (EE) of LNP-formulated HPV16 L1 mRNA were characterized. Particle size, PDI, and zeta potential were measured by dynamic light scattering (DLS) using Zetasizer Nano ZS (Malvern Panalytical, Malvern, UK). EE was determined using ribogreen assay.

### 2.7 Immunization and sample collection

BALB/c female mice (6 weeks old) received intramuscular injections of either GARDASIL^®^ 9 (MSD, New Jersey, USA) or LNP–encapsulated mRNA encoding HPV16 L1. Immunizations were administered at a dose of 1, 5, 10 µg on days 0, 14, and 28, while control animals were injected with PBS. Blood samples were obtained every two weeks via retro-orbital collection under inhalational anesthesia using 1–5% isoflurane delivered with oxygen. Two weeks following the final dose, animals were exposed to HPV pseudovirus (PsV) and splenocytes were collected. All animal procedures were reviewed and approved by the Institutional Animal Care and Use Committee (IACUC) of the Center of Animal Care and Use (CACU), Lee Gil Ya Cancer and Diabetes Institute (Incheon, Korea) (approval number: LCDI-2024-0080).

### 2.8 HPV16 PsV challenge in mice

HPV PsV infection procedure was adapted from a previously described protocol (30). This model has been previously validated as a quantitative platform for assessing *in vivo* protective efficacy of HPV vaccine candidates (31–33). PsVs were provided by Yonsei University (Seoul, Korea). Mice received 100 μL of 30 mg/mL Depo-Provera (Sigma-Aldrich, St. Louis, USA) subcutaneously. PsVs were mixed 1:1 (v/v) with 3% carboxymethylcellulose (CMC). Three days later, mice were anesthetized with Isoflurane, and 20 μL of PsVs-3% CMC mixture was administered intravaginally using positive-displacement pipette. A pap-brush was inserted into the vagina and rotated firmly 10–20 times until light reddish blood appeared to induce epithelial abrasion, after which an additional 20 μL of the mixture was administered intravaginally. Three days post-infection, 20 μL of 7.8 mg/mL firefly luciferin (Revvity, Waltham, USA) was administered intravaginally, and infection was monitored using *in vivo* imaging system (IVIS) (Spectral Instruments Imaging, Tucson, USA). Images were analyzed using AURA v4.0.8.

### 2.9 Virus-like particle ELISA

VLPs were provided by Yonsei University (Seoul, Korea). ELISA plates (Greiner Bio-One, Kremsmünster, Austria) were coated with 100 μL/well of HPV16 VLP diluted in PBS at a concentration of 25 ng/well and incubated overnight at 4 °C. After three washes with PBS, wells were blocked with 150 μL/well of 1% BSA/PBS and incubated at 37 °C for 2 hours. Following three additional PBS washes, serum samples diluted in 1% BSA/PBS were added at 100 μL/well and incubated overnight at 4 °C. Mouse sera were diluted 1:100, and standard serum (10/140, NIBSC, Potters Bar, UK) was diluted 1:200 and serially diluted 2-fold for 8 points. After four washes with PBST, HRP-conjugated secondary antibodies diluted in PBST were added at 100 μL/well and incubated at 37 °C for 2 hours. Mouse IgG secondary antibody (SouthernBiotech, Birmingham, USA) was diluted 1:5,000, while mouse IgG1 and IgG2a secondary antibodies (Cell Signaling Technology, Danvers, USA) and human secondary antibody (Cell Signaling Technology, Danvers, USA) were diluted 1:3,000. After five PBS washes, 100 μL/well of TMB substrate solution was added and incubated at room temperature in the dark for 15 minutes. The reaction was terminated with 50 μL/well of TMB stop solution, and absorbance was measured at 450 nm using a microplate reader. Data were acquired using SoftMax Pro v5.4.1. For quantification of binding antibody levels, the standard serum was assigned concentration of 1,000 AU/mL, and serial dilutions were used to generate standard curve. Standard curve was fitted using a sigmoidal, four-parameter logistic (4PL) model with X defined as log(concentration) in GraphPad Prism v8.0.2. Sample antibody concentrations were calculated by interpolating the corresponding OD values from the fitted standard curve and expressed as AU/mL.

### 2.10 Pseudovirus-based neutralization assay

HEK293TT cells were seeded in 96-well flat-bottom plates at 3×10^4^ cells/well in 100 μL of culture media (DMEM supplemented with 10% FBS, 1% NEAA, 1% Glutamax, 1% antibiotic-antimycotic) and incubated for 5 hours at 37 °C with 5% CO_2_. Serum samples were diluted 1:40 in 8-point, 4-fold series, while standard serum (10/140, NIBSC, Potters Bar, UK) was diluted 1:10 in 8-point, 4-fold series. PsV was diluted in media to 300 TCID50/well. Serum and PsV mixtures (1:4, v/v) were incubated at 4 °C for 1 hour, then added to HEK293TT cells. Following 72 hours of incubation at 37 °C, 5% CO_2_, 100 μL of media was removed and replaced with 100 μL of ONE-Glo luciferin (Promega, Madison, USA) diluted 1:3 in distilled water. Plates were incubated at room temperature for 3 minutes, resuspended 10–15 times to detach cells, and 150 μL transferred to 96-well flat-bottom black plates, avoiding bubbles. Luciferase signals were measured using luminometer (measurement interval, 0.3 s). Neutralization curves were fitted using four-parameter logistic (4PL) nonlinear regression model with variable slope, and IC50 values were calculated from the fitted curves using GraphPad Prism v8.0.2. Neutralization (%) was calculated using the following formula.


$$\text{Neutralization}\left(\%\right)=100-\left[\frac{\text{RLU}\left(\text{sample}\right)-\text{RLU}\left(\text{Mock}\right)}{\text{RLU}\left(\text{PsV only}\right)-\text{RLU}\left(\text{Mock}\right)}\text{ X }100\right]$$

IU/mL values were calculated using the same standard curve-based method described for ELISA, with antibody concentrations expressed in IU/mL rather than AU/mL.

### 2.11 Intracellular cytokine staining

Mouse splenocytes were prepared by crushing spleens through 70 μm cell strainers and washed with PBS. Cells were centrifuged at 1600 rpm for 5 minutes, resuspended, and RBCs lysed with pre-warmed lysis buffer at 37 °C for 5 minutes. Cells were resuspended in RPMI with 10% FBS and counted. For antigen-specific intracellular cytokine analysis, splenocytes (1×10^6^ cells/well) were stimulated with HPV16 L1 peptides (PM-HPV16-L1, JPT Peptide Technologies, Berlin, Germany) 1 μg/mL and Golgi-plug 1 μL/mL for 6 hours at 37 °C with 5% CO_2_. Cells were stained for surface markers CD4-FITC and CD8-PE (BD Biosciences, Franklin Lakes, USA), followed by intracellular cytokines IFNγ-BV421 (BioLegend, San Diego, USA), TNFα-APC-Cy7, and IL-2-PerCP-Cy5.5 (BD Biosciences, Franklin Lakes, USA) using Cytofix/Cytoperm™ Plus kit (BD Biosciences, Franklin Lakes, USA). All antibodies were diluted at 1:100, and staining was performed at 4 °C for 30 minutes. Data were acquired using BD LSRII and analyzed with FlowJo v10 (BD Biosciences, Franklin Lakes, USA).

### 2.12 Enzyme-linked immunospot

Mouse IFN-γ ELISpot assays were performed according to the manufacturer’s instructions (R&D Systems, Minnesota, USA). Briefly, ELISpot plates were pre-equilibrated by adding 200 μL/well of RPMI supplemented with 10% FBS and 1% antibiotic–antimycotic solution and incubating at room temperature for 20 minutes. After removal of the medium, mouse splenocytes were seeded at 1×10^5^ cells/well in 50 μL and stimulated with HPV16 L1 peptides (JPT Peptide Technologies, Berlin, Germany) 1 μg/mL at 37 °C in 5% CO_2_ for 20 hours. Following incubation, plates were washed four times with 300 μL/well of wash buffer. After the final wash, plates were inverted and tapped dry on paper towels. Streptavidin-AP concentrate A (100 μL/well) was added and incubated overnight at 4 °C. Plates were then washed four times with wash buffer, followed by the addition of BCIP/NBT substrate (100 μL/well). Plates were incubated for 1 hours at room temperature in the dark. After terminating the reaction, plates were washed four times with distilled water. Plates were inverted to remove residual liquid and the plastic underdrain was removed. After complete air-drying at room temperature, spots were analyzed using an ELISpot reader (AID autoimmun diagnostika gmbh, Straβberg, Germany). For analysis, unstimulated wells were used to determine background response. The mean number of spot-forming units (SFU) in the corresponding unstimulated wells was subtracted from SFU counts of antigen-stimulated wells, and the resulting background-subtracted values were used for data presentation.

### 2.13 Statistical analysis

All statistical analyses were performed using GraphPad Prism v8.0.2. Two-group comparisons were conducted using Mann-Whitney U test, while multiple groups were analyzed by Kruskal-Wallis test with Dunn’s *post hoc* test. Statistical significance was defined as P< 0.05. Levels of significance were indicated as follows: ns, not significant, *P< 0.05, **P< 0.01, ***P< 0.001 and ****P< 0.0001. Data were presented as mean ± standard error of the mean (SEM) for line graphs and as median for dot plots.

### 2.14 Use of AI-assisted language editing

ChatGPT (OpenAI, GPT-5.2) was used for English language translation.

## 3 Results

### 3.1 Characterization and validation of HPV16 mRNA vaccine

An mRNA vaccine encoding HPV16 L1 was designed. The sequence was cloned into IVT vector CUK3-1 (**Figure 1A**). mRNA was synthesized via IVT. Denaturing agarose gel electrophoresis revealed a band corresponding to the expected size (~2 kb). After LiCl precipitation, the increased band intensity was consistent with the removal of contaminants other than mRNA (**Figure 1B**). Following double-stranded RNA removal, the integrated density value decreased from 10% to 0%. The reduced signal in dot blot suggested that predominantly single-stranded mRNA remained (**Figure 1C**). To assess protein expression from the synthesized mRNA, HEK293TT cells were transfected and analyzed by western blot. A distinct band corresponding to the expected molecular weight of L1 (~56 kDa) was observed in mRNA-transfected samples (**Figure 1D**). For quality control, the physicochemical properties of HPV16 L1 mRNA encapsulated in LNPs were characterized by measuring particle size, polydispersity index (PDI), zeta potential, and encapsulation efficiency (EE). The average particle size was 91.08 ± 10.06 nm, with PDI of 0.2 ± 0.02. LNPs exhibited a positive zeta potential of 19.47 ± 3.6 mV and mRNA encapsulation efficiency of 91.35 ± 1.35% (**Figure 1E**). These results demonstrated efficient encapsulation of HPV16 L1 mRNA within LNP formulation (34).

**Figure 1:**
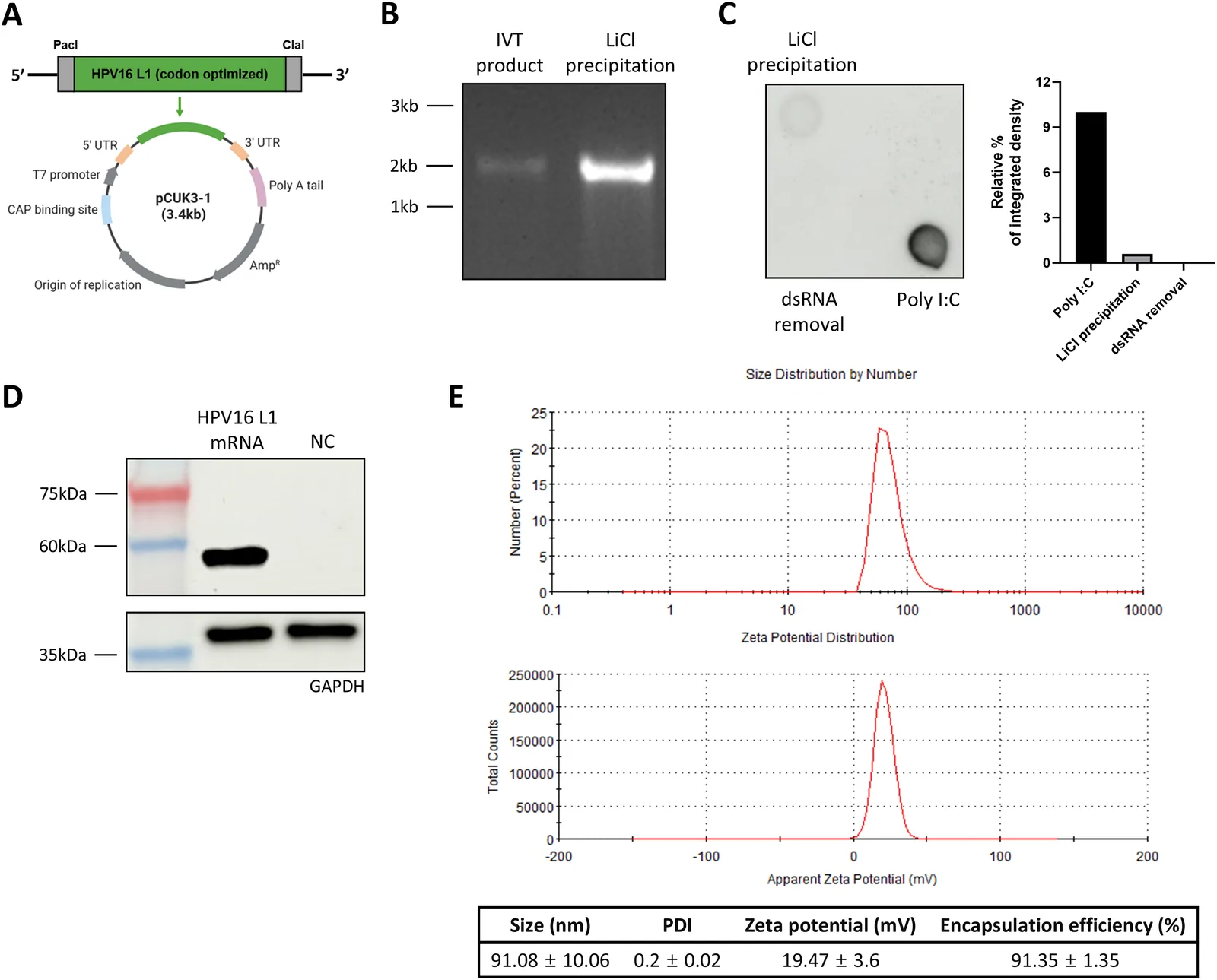
Construction and validation of HPV16 mRNA vaccine. **(A)** Characterization of vector for *in vitro* transcription (IVT). The vector contains T7 promoter, 5′ and 3′ UTRs, a poly A tail, and CAP-binding site for IVT. **(B)** Denaturing agarose gel showed a clear 2 kb mRNA band with increased intensity after lithium chloride (LiCl) precipitation. **(C)** Dot blot analysis confirmed effective removal of double-stranded RNA (dsRNA), indicating high-purity single-stranded mRNA. **(D)** Western blot analysis of HPV16 L1 expression in cell lysates 24 hours post-transfection with HPV16 mRNA. Non-transfected cell lysates served as NC. GAPDH was used as a loading control. **(E)** Physicochemical characterizations of mRNA-lipid nanoparticle (LNP). For quality control, the particle size, polydispersity index (PDI) and zeta potential of mRNA-HPV16 encapsulated in LNPs were measured by dynamic light scattering (DLS). Encapsulation efficiency (EE) was assessed using ribogreen assay. Data were presented as mean of triplicate measurements ± SEM. The schematic illustration was created using biorender.com.

### 3.2 Definition of protection threshold and immune correlates of protection based on GARDASIL^®^ 9

To define a protection threshold, the licensed prophylactic vaccine GARDASIL^®^ 9 was serially diluted from 10² to 10^4^ and administered to BALB/c mice three times at two-week intervals. Following HPV16 PsV challenge, protective efficacy was assessed using IVIS. Bioluminescence imaging revealed inconsistent signal intensities among individual animals in the group immunized with GARDASIL^®^ 9 diluted to 10^4^ (**Figure 2A**). Quantification of radiance within the region of interest (ROI) similarly demonstrated substantial variability (**Figure 2B**), indicating that complete protection was not consistently achieved at this dilution. In contrast, mice receiving GARDASIL^®^ 9 at dilutions up to 10^3^ exhibited markedly reduced bioluminescence signals compared with Mock group, and consistent protection was observed in all animals.

**Figure 2:**
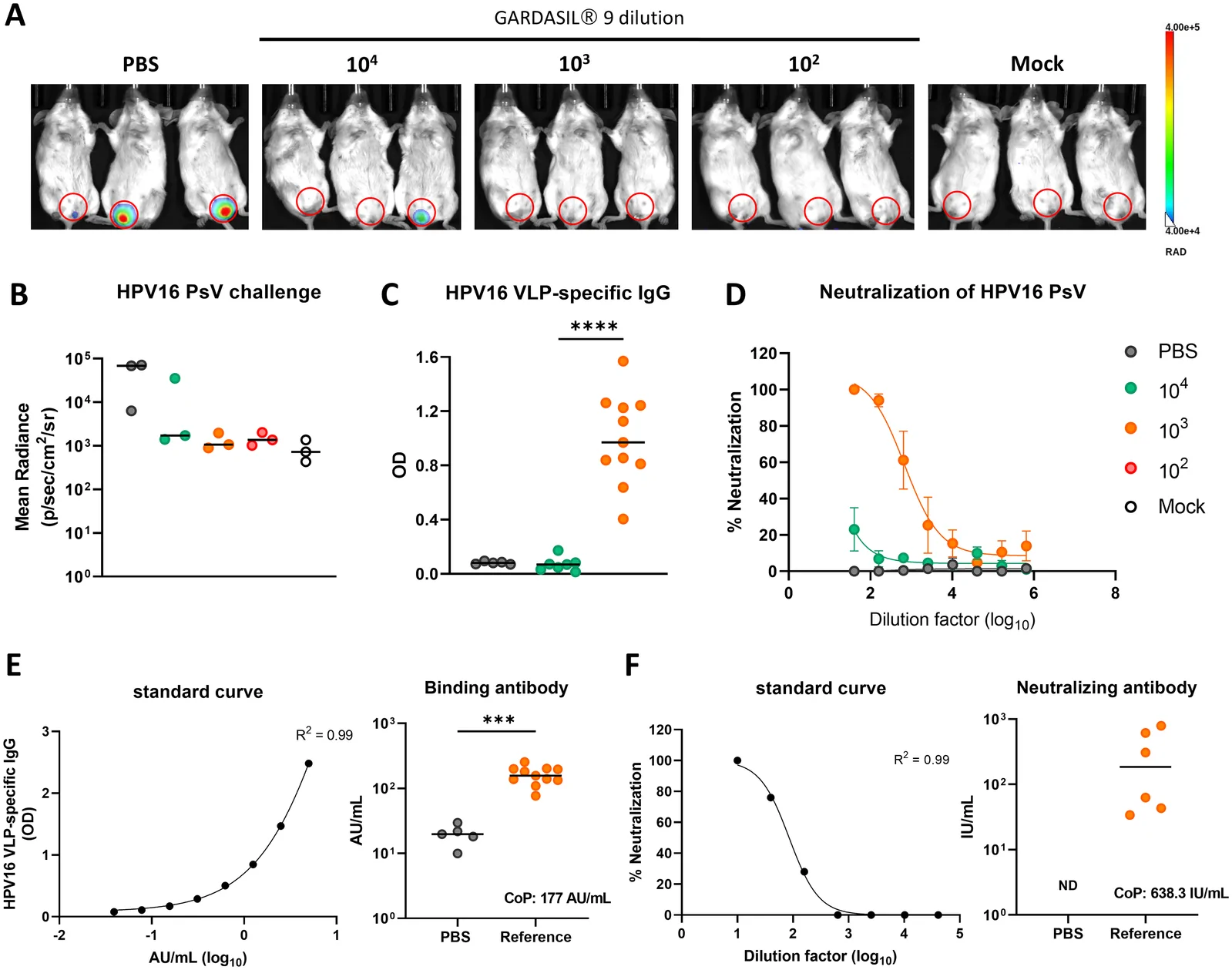
The protection threshold was determined based GARDASIL^®^ 9. BALB/c mice were immunized intramuscularly three times at two-week intervals with PBS or GARDASIL^®^ 9 at dilutions ranging from 10^2^ to 10^4^. Two weeks after the final immunization, blood samples were collected and mice were challenged intravaginally with HPV16 pseudovirus (PsV). Mock was not subjected to PsV challenge. Three days after challenge, bioluminescence imaging was performed using *in vivo* imaging system (IVIS). **(A)** Bioluminescence images of each group. Representative images from one of three independent experiments were shown. Red circles indicated the region of interest (ROI) used for signal quantification. **(B)** Quantification of bioluminescence signals measured by IVIS imaging. Signal intensity was determined as mean radiance within ROI. **(C)** HPV16 VLP–specific IgG (OD values) measured by ELISA in mice immunized with PBS or GARDASIL^®^ 9 diluted to 10^3^ and 10^4^. **(D)** HPV16 pseudovirus–based neutralization assay (PBNA) results shown as percent neutralization curves using sera from mice immunized with PBS or GARDASIL^®^ 9 diluted to 10^3^ and 10^4^. The antibody titer at the 10^3^ dilution point of GARDASIL^®^ 9 was used as Reference for efficacy assessment. **(E)** A standard curve for calculating binding antibody titers was generated by plotting ELISA-derived OD values against arbitrary unit (AU/mL) using standard serum. Measured OD values were converted to AU/mL using a standard curve. **(F)** A standard curve for calculating neutralizing antibody titers was derived by plotting PBNA-derived neutralization percentages against dilution factors using standard serum. IC50 titers were interpolated from the standard curve, and the corresponding international unit (IU/mL) of the original samples were calculated. Each dot represented an individual mouse. Data were pooled from three independent experiments. Sample sizes for each group were indicated in the graph. Data were presented as mean ± standard error of the mean (SEM) for line graphs and as median for dot plots. Statistical analyses were performed using Mann-Whitney U (***P< 0.001 and ****P< 0.0001). ND, no data.

To further confirm complete protection at 10^3^ dilution, VLP-binding and neutralizing antibody titers were measured. In 10^4^ dilution group, binding antibodies were barely detectable, and no neutralizing activity was observed. In contrast, 10^3^ dilution group induced binding antibodies as well as measurable neutralizing activity (**Figures 2C, D**). These findings suggested that 10^3^ dilution of GARDASIL^®^ 9 represented the minimum effective dilution capable of conferring complete protection.

Accordingly, a serum dilution of 10^3^ was defined as the minimum threshold required for protection, and the corresponding antibody titers were established and used as Reference for evaluating the efficacy of HPV vaccine candidates. To this end, assays were standardized using a concentration-defined standard serum to generate standard curves, thereby enabling conversion of assay readouts into quantitative antibody concentrations. Using this approach, binding antibody titers were quantified in arbitrary units (AU), and neutralizing antibody titers were expressed in international units (IU) (**Figures 2E, F**). Therefore, the median antibody titer derived from sera of mice immunized with GARDASIL^®^ 9 diluted to 10^3^ was used as Reference and was defined as CoP in this study.

### 3.3 HPV16 mRNA induces *in vivo* protection and robust humoral immune responses

Given the limited number of studies evaluating HPV L1 mRNA–based vaccines, immunogenicity and protective efficacy were initially examined under a high-dose vaccination to allow robust immune responses to be assessed. Based on existing evidence demonstrating protective effects following immunization with 10 μg of a nucleic acid–based HPV L1 DNA vaccine, we selected 10 μg as the initial dose for HPV L1 mRNA vaccine (35). Synthesized HPV16 mRNA was encapsulated in LNPs and administered to BALB/c mice at a dose of 10 μg, three times at two-week intervals. PBS and undiluted GARDASIL^®^ 9 were included as control groups. Binding antibody titers (AU/mL) and neutralizing antibody titers (IU/mL) were quantified using serum samples collected two weeks after the final immunization (Day 42). *In vivo* protection was assessed by IVIS following HPV16 PsV challenge. Both HPV16 mRNA and GARDASIL^®^ 9 groups exhibited minimal to undetectable bioluminescent signals, whereas PBS group showed robust luciferase activity (**Figure 3A**). Consistent with the bioluminescence imaging results, quantitative analysis revealed reduced assay readouts in both HPV16 mRNA and GARDASIL^®^ 9 groups compared with PBS group, indicating protection against HPV16 PsV (**Figure 3B**).

**Figure 3:**
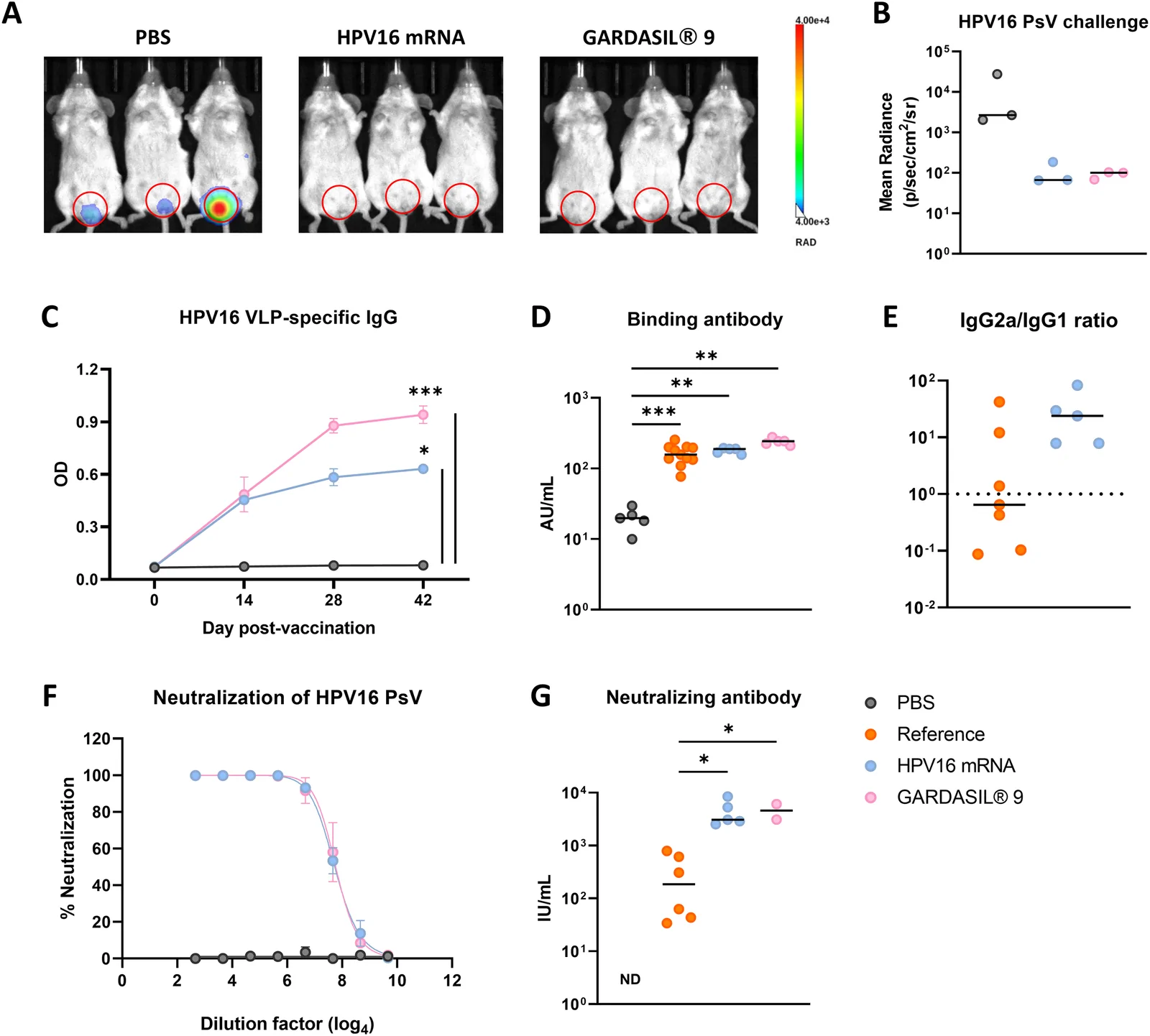
Protective efficacy and immunogenicity of HPV16 mRNA. BALB/c mice were immunized intramuscularly three times at two-week intervals with PBS, HPV16 mRNA or GARDASIL^®^ 9 (n = 5). Mice were challenged intravaginally with HPV16 pseudovirus (PsV) two weeks after the final immunization. Blood samples were collected at two-week intervals. Three days post-challenge, bioluminescence signals were acquired using *in vivo* imaging system (IVIS). **(A)** Representative bioluminescence images were shown. IVIS imaging was performed on 3 of the 5 mice in each group. The region of interest (ROI) used for signal quantification was outlined by red circles. **(B)** Bioluminescence intensity was expressed as mean radiance within ROI. **(C)** Time-dependent increase in HPV16 VLP-specific IgG (OD values) measured by ELISA. **(D)** HPV16 VLP-specific IgG titers were converted to AU/mL using standard curve. **(E)** HPV16 VLP–specific IgG2a/IgG1 ratios measured by ELISA. Ratio > 1, IgG2a dominant; ratio< 1, IgG1 dominant. **(F)** Neutralization curves against HPV16 PsV were generated using pseudovirus-based neutralization assay (PBNA). **(G)** HPV16 PsV specific neutralizing antibody titers were converted to IU/mL using standard curve. Reference group consisted of mice immunized with 10^3^ dilution of GARDASIL^®^ 9. Each dot represented an individual mouse. Data from Reference group were pooled from three independent experiments. Sample sizes for each group were indicated in the graph. Data were presented as mean ± standard error of the mean (SEM) for line graphs and as median for dot plots. Statistical analyses were performed using Kruskal-Wallis test with Dunn’s *post hoc* test (*P< 0.05, **P< 0.01 and ***P< 0.001). ND, no data.

Antibody responses elicited by HPV16 mRNA increased progressively with successive immunizations, whereas no appreciable elevation in antibody levels was observed in PBS group (**Figure 3C**). Measurement of HPV16 VLP-binding antibodies using the standardized assay revealed that HPV16 mRNA group consistently exhibited a robust humoral response, characterized by high binding antibody titers. These responses were comparable to those induced by GARDASIL^®^ 9 group and were also similar to IgG levels of Reference group. In contrast, only background-level signals were detected in PBS group (**Figure 3D**). Analysis of IgG subclasses revealed that HPV16 mRNA immunization resulted in increased IgG2a/IgG1 ratios, consistent with the induction of Th1 immune response. In contrast, Reference group showed variable responses among individual animals (**Figure 3E**).

Neutralizing activity against HPV16 PsV were evaluated using PBNA. Both HPV16 mRNA and GARDASIL^®^ 9 groups exhibited strong and nearly equivalent neutralizing responses, whereas no detectable neutralization was observed in PBS group (**Figure 3F**). Consistent with these findings, calculation of neutralizing antibody titers demonstrated that HPV mRNA group achieved high neutralization titers comparable to those induced by GARDASIL^®^ 9 group and exceeding the predefined neutralizing antibody level of Reference group (**Figure 3G**).

### 3.4 HPV16 mRNA is effective in inducing T cell responses in the spleen

mRNA vaccines have been reported to induce innate immune activation and elicit both humoral and cellular immune responses (12, 13, 26). Two weeks after the final immunization, HPV16 L1–specific CD4^+^ and CD8^+^ T cell responses were assessed in splenocytes. In HPV16 mRNA group, CD4^+^ T cells producing IFN-γ (0.30%), TNF-α (0.66%), and IL-2 (0.31%) were detected at markedly higher frequencies than those observed in PBS group (0.007%, 0.42%, and 0.23%, respectively) (**Figure 4A**). Consistently, CD8^+^ T cells from HPV16 mRNA group exhibited substantially enhanced cytokine production, including IFN-γ (16.7%), TNF-α (8.64%), and IL-2 (0.95%), compared with PBS group (0.094%, 0.28%, and 0.12%, respectively) (**Figure 4B**). Overall, cytokine-secreting CD4^+^ and CD8^+^ T cell populations were detected exclusively in HPV16 mRNA group, whereas such responses were not observed in either PBS or GARDASIL^®^ 9 groups (**Figures 4C, D**).

**Figure 4:**
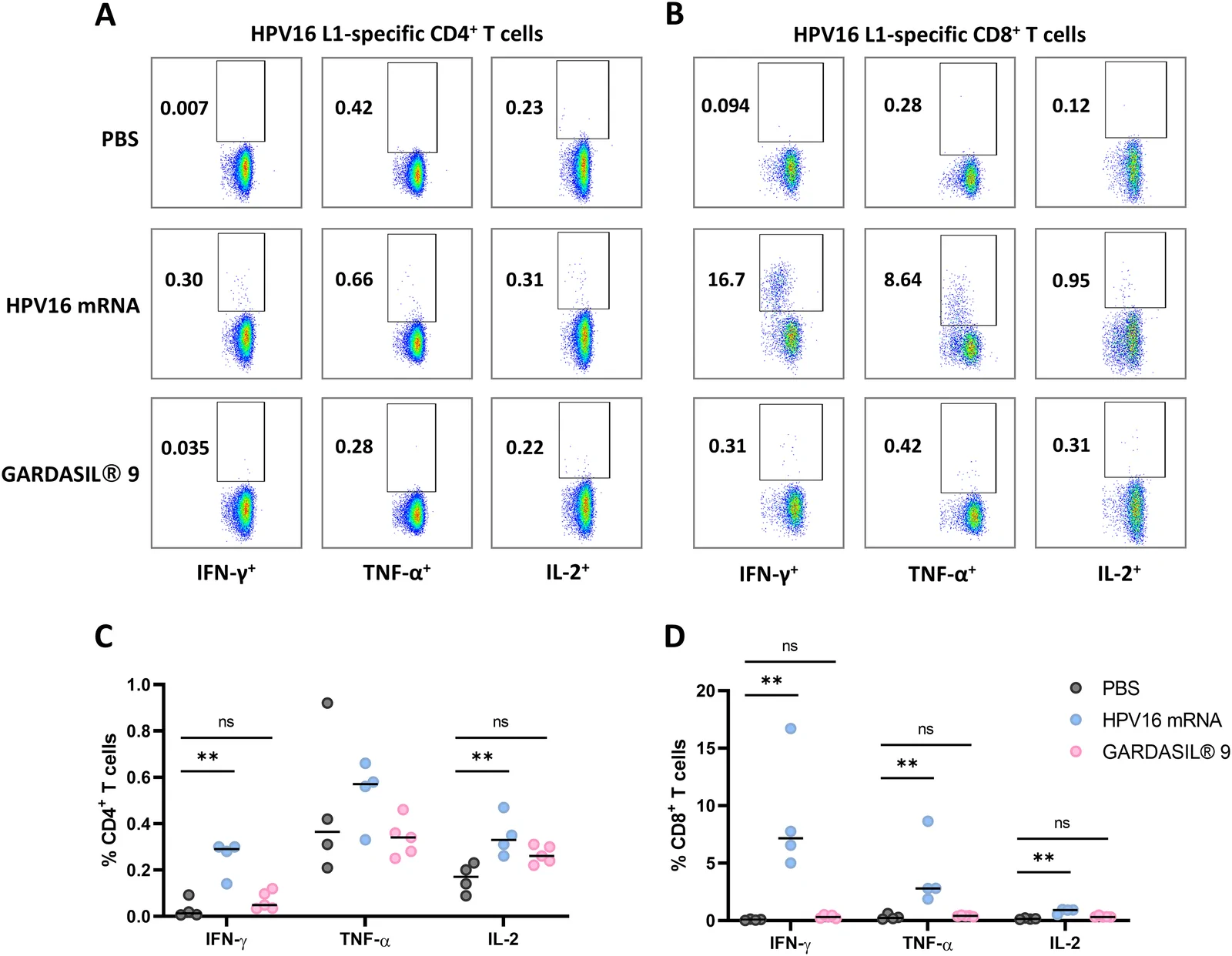
HPV16-specific T-cell responses in spleen induced by HPV16 mRNA immunization. Representative flow cytometry plots showing % of CD4 T cells **(A)** and CD8 T cells **(B)** producing IFN-γ, TNF-α, and IL-2 following HPV16 L1 peptide stimulation. Total % of CD4 T cells **(C)** and CD8 T cells **(D)** producing cytokines. Each dot represented an individual mouse. Data were presented as median. Statistical analyses were performed using Kruskal-Wallis test with Dunn’s *post hoc* test (ns = not significant and **P< 0.01).

### 3.5 Dose-dependent protective efficacy and immunogenicity of HPV16 mRNA vaccination

Because high doses of mRNA-LNP vaccines can induce inflammatory responses, we evaluated whether protective immunity is maintained following dose reduction of HPV16 mRNA vaccine in immunized mice (36). Following HPV16 PsV challenge, bioluminescence imaging using IVIS revealed no detectable signals in mice vaccinated with 1, 5, or 10 μg of HPV16 mRNA (**Figure 5A**). Quantitative analysis also demonstrated near-complete protection against HPV16 PsV across all mRNA dose groups (**Figure 5B**). Binding antibody titers showed no substantial differences between Reference and mRNA groups, nor were marked variations observed across different mRNA dose levels (**Figure 5C**). Neutralizing antibody titers in HPV16 mRNA 1 μg group were comparable to those of Reference group, whereas higher titers were induced in mice vaccinated with 5 or 10 μg doses (**Figures 5D, E**). Although neutralizing antibody titers elicited by 1 μg dose were lower than those observed at higher doses, complete protection against HPV16 PsV infection was achieved. (**Figures 5A, B**). IgG2a/IgG1 ratios remained elevated regardless of HPV16 mRNA dose, consistent with Th1 immune response. Responses in Reference group, however, were variable among individual animals (**Figure 5F**).

**Figure 5:**
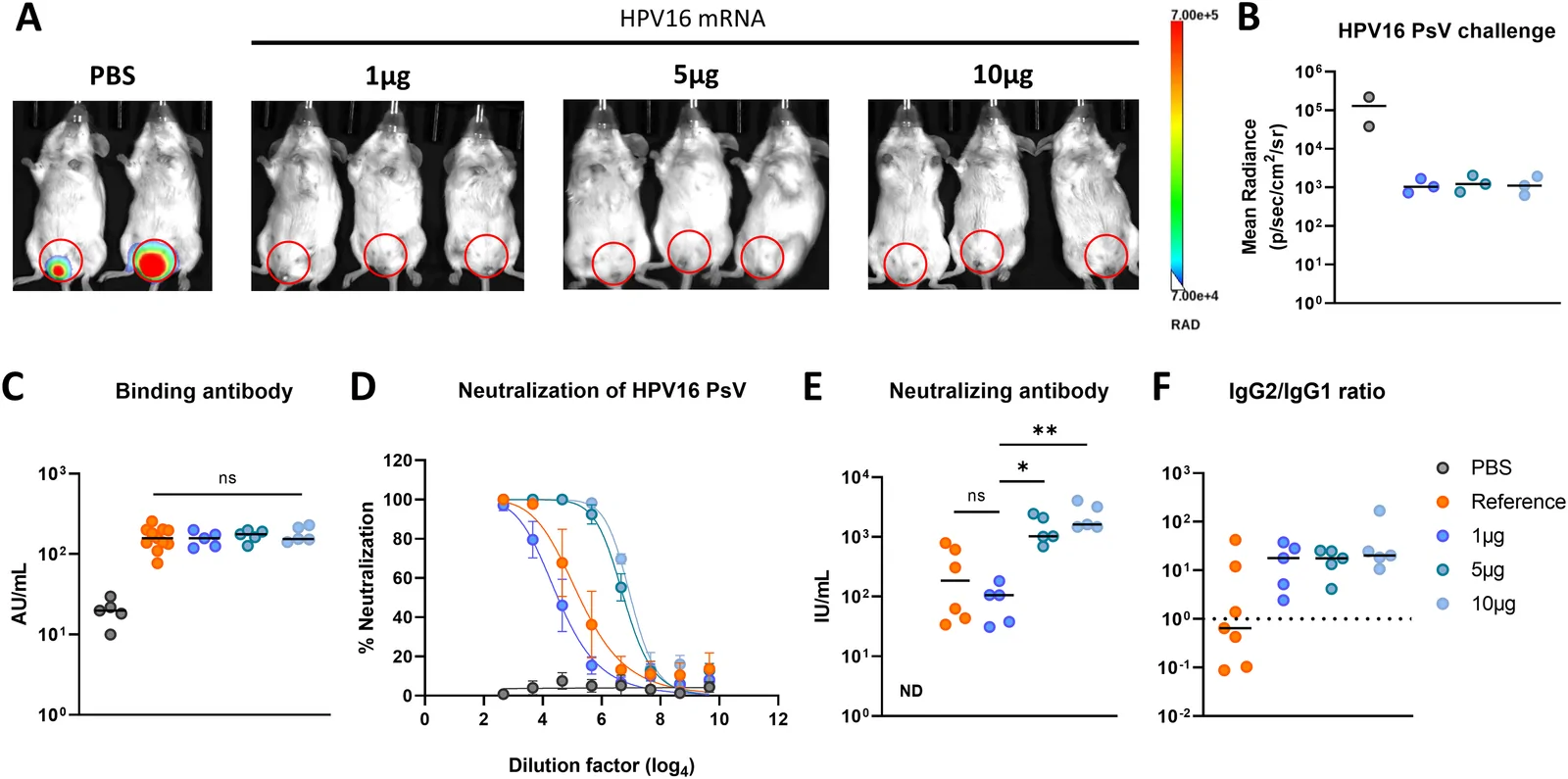
Protective efficacy and immunogenicity of HPV16 mRNA vaccine at low doses. BALB/c mice were immunized intramuscularly three times at two-week intervals with PBS or HPV16 mRNA at doses of 1, 5, or 10 μg. Two weeks after the final immunization, blood samples were collected and mice were challenged intravaginally with HPV16 pseudovirus (PsV). Bioluminescence signals were acquired using an *in vivo* imaging system (IVIS) at 3 days post-challenge. **(A)** Representative IVIS bioluminescence images from each group are presented. Imaging was conducted on 2 or 3 of the 5 mice per group. Red circles indicated the regions of interest (ROIs) used for signal analysis. **(B)** Quantified bioluminescence signals were shown as mean radiance values measured within the ROIs. **(C)** HPV16 VLP–specific IgG titers were determined by ELISA and converted to AU/mL using standard curve. **(D)** Neutralization curves against HPV16 PsV were generated using pseudovirus-based neutralization assay (PBNA). **(E)** HPV16 PsV specific neutralizing antibody were converted to IU/mL using standard curve. **(F)** HPV16 VLP –specific IgG2a/IgG1 ratios measured by ELISA in mice immunized with HPV16 mRNA at the indicated doses. Ratio > 1, IgG2a dominant; ratio< 1, IgG1 dominant. Reference group consisted of mice immunized with 10^3^ dilution of GARDASIL^®^ 9. Each dot represented an individual mouse. Data from Reference group were pooled from three independent experiments. Sample sizes for each group were indicated in the graph. Data were presented as mean ± standard error of the mean (SEM) for line graphs and as median for dot plots. Statistical analyses were performed using Kruskal-Wallis test with Dunn’s *post hoc* test (ns = not significant, *P< 0.05 and **P< 0.01). ND, no data.

### 3.6 Low-dose HPV16 mRNA induces IFN-γ secretion and cytokine-producing T cell responses in the spleen

Although neutralizing antibody titers induced in HPV16 mRNA 1 μg group were slightly lower than those in Reference group (**Figures 5D, E**), complete protection against PsV challenge was observed (**Figures 5A, B**). Notably, given the antigen-specific T cell responses identified in this study (**Figure 4**), cellular immune responses may have compensated for the relatively lower neutralizing antibody titers and contributed to protection. Based on this observation, T cell responses in the low-dose immunization group were evaluated. In addition, we assessed the secretion of IFN-γ, a key cytokine of Th1 immunity. Following stimulation with HPV16 L1 peptides, IFN-γ–secreting cells were observed only in HPV16 mRNA 1 μg group (**Figure 6A**). Consistent with this result, increased frequencies of cytokine-secreting activated T cells were observed in HPV16 mRNA 1 μg group compared with Reference group. In CD4 T cells, TNF-α (0.27%) and IL-2 (0.36%) were detected in HPV16 mRNA 1 μg group, compared with 0.08% and 0.15% in Reference group, respectively. In CD8 T cells, cytokine secretion levels were 2.31% and 1.02% in HPV16 mRNA 1 μg group, compared with 0.016% and 0.3% in Reference group (**Figures 6B, C**).

**Figure 6:**
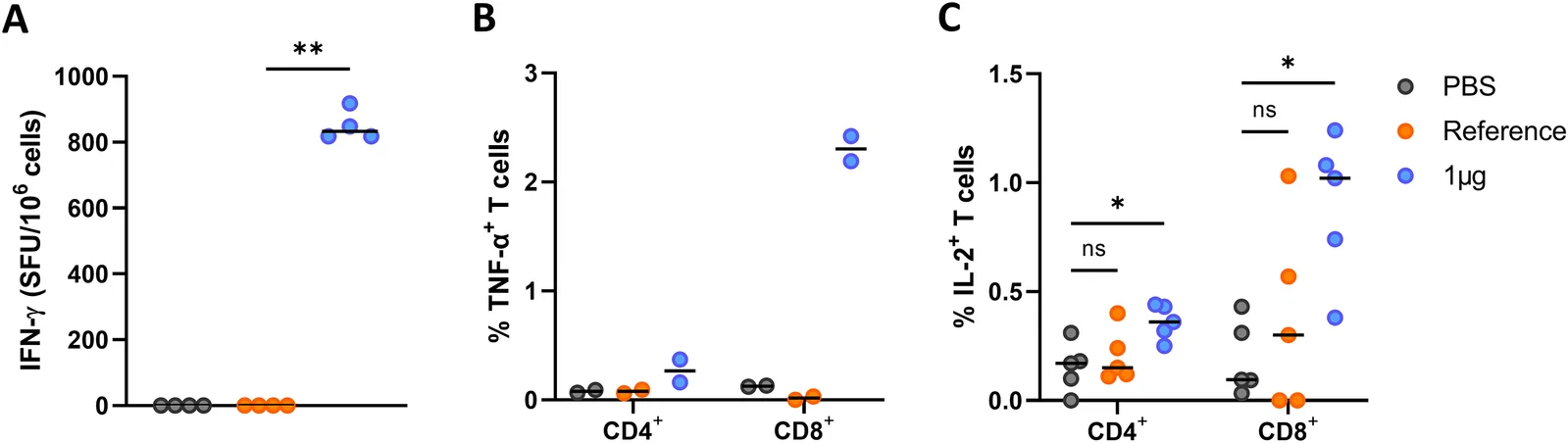
HPV16-specific cytokine responses in the spleen following low-dose HPV16 mRNA immunization. Splenocytes were isolated from immunized mice and stimulated with HPV16 L1 peptide. IFN-γ producing cells were quantified by ELISpot **(A)**, and results were expressed as spot-forming units (SFU) per well using splenocytes from two individual mice with each condition tested in duplicate wells. The frequencies of TNF-α– **(B)** and IL-2– **(C)** expressing T cells were analyzed by flow cytometry. Each dot represented an individual mouse. Data were presented as median. Statistical analyses were performed using Mann-Whitney U test or Kruskal-Wallis test with Dunn’s *post hoc* test (ns = not significant, *P< 0.05 and **P< 0.01).

## 4 Discussion

In this study, evaluation assays were established to determine the efficacy of novel HPV vaccines and were applied to an mRNA vaccine candidate, thereby validating the reliability of the proposed CoP and supporting the feasibility of mRNA-based HPV vaccine development. To achieve this, analytical methods for the quantitative measurement of antibody responses were standardized, and HPV16 mRNA vaccine was designed. Using GARDASIL^®^ 9 as Reference, we defined the minimum antibody level required for protection and applied this threshold to evaluation of HPV16 mRNA vaccine. Notably, a meaningful level of protection was observed even in the low-dose group (1 μg). Furthermore, antibody responses in this group were comparable to the defined protective level, supporting the reliability of the proposed CoP.

All currently licensed prophylactic HPV vaccines are recombinant protein formulations based on L1 VLPs (3, 7). These particles structurally resemble native virions but lack viral genomic material and therefore cannot replicate, which enhances their safety (37). In addition, VLPs assemble into highly ordered, repetitive surface arrays that efficiently cross-link B cell receptor (BCR), thereby promoting robust immunogenicity (38). Despite these immunological advantages, the production of highly purified and structurally stable VLPs requires complex manufacturing processes to minimize contamination and ensure product consistency, thereby increasing production costs and contributing to lower vaccination coverage in low- and middle-income countries (9, 10, 39, 40). In contrast, mRNA vaccines require a relatively short timeline for design and production, and their manufacturing processes can be efficiently scaled for large-scale synthesis and purification, enabling rapid vaccine development and sustained supply (13, 14). Moreover, because mRNA is transiently expressed and rapidly degraded *in vivo*, it does not integrate into the host genome, thereby minimizing the risk of insertional mutagenesis and reinforcing its safety (15, 41). Therefore, mRNA vaccines have the potential to overcome a major limitation of VLP-based vaccines, specifically high manufacturing costs, and thereby expand global access to vaccination.

HPV L1 is a major structural protein that plays an important role in viral entry and has been widely utilized as a key antigen for prophylactic vaccine development (5, 6). Accordingly, we constructed mRNA vaccine encoding HPV16 L1 in this study. Because neutralizing antibodies elicited by HPV L1-based VLP and capsomere are known to predominantly recognize conformational epitopes of L1, it is important to determine not only whether the L1 mRNA is translated into protein but also whether the expressed L1 protein assembles into an appropriate higher-order structure (22, 23). However, in this study, Western blotting confirmed the expression of L1 protein, primarily in its monomeric form, but did not directly demonstrate the formation of higher-order structures such as VLP or capsomere. Nevertheless, significant L1-specific neutralizing antibody responses were detected in sera from mice immunized with HPV16 mRNA by PBNA. These findings indirectly support the possibility that the expressed L1 protein adopted structural features sufficient to retain antigenicity and elicit antibodies capable of recognizing conformational epitopes.

To establish a quantitative protection threshold, we used an *in vivo* HPV PsV challenge model that has been validated in previous studies (31–33). Because HPV is a species-specific virus, this model does not fully recapitulate natural HPV infection. However, in this study, the model was used not to reproduce the natural course of HPV infection, but to determine whether vaccine-induced antibody responses can neutralize HPV *in vivo* and confer protective efficacy against infection. By analyzing sera from mice immunized with serially diluted GARDASIL^®^ 9 in this challenge model, we defined binding and neutralizing antibody titers associated with complete protection, as indicated by the absence of bioluminescent signals following challenge.

Because different host-specific secondary antibodies and dilution factors were used for the analysis of human and mouse sera, ELISA-derived antibody levels expressed in AU/mL have limitations for direct quantitative comparison across species or for conversion to standardized IU/mL, as is possible for PBNA. Therefore, AU/mL values should be interpreted as assay- and model-dependent measures of antigen-specific binding antibody responses rather than absolute antibody concentrations that can be directly compared across species. Nevertheless, considering the primary objective of this study to establish a standardized evaluation framework, ELISA-based AU/mL measurements remain valuable for assessing and comparing antigen-specific binding antibody responses under standardized experimental conditions during preclinical evaluation. In contrast, PBNA provides a functional measure of virus-neutralizing activity and is less affected by differences in host origin, as it does not require host-specific secondary antibodies.

The results showed that binding antibody titers were not significantly different between CoP and HPV16 mRNA-immunized groups, supporting their utility for confirming the induction of antigen-specific immune responses. In contrast, neutralizing antibody titers were induced at higher levels than Reference and exhibited dose-dependent differences, suggesting more direct association with functional protective immunity. Accordingly, neutralizing antibody responses may provide a more functionally relevant measure for assessing vaccine-induced protection, whereas binding antibody measurements can serve as an initial indicator of immunogenicity. Given their analytical simplicity, reproducibility, and suitability for high-throughput analysis, binding antibody assays may be particularly useful for early-stage screening, while PBNA can provide subsequent functional validation of vaccine-induced neutralizing activity. Thus, incorporating both measures in a complementary manner may provide a practical and comprehensive strategy for evaluation of HPV vaccine candidates.

IgG subclass profiling was performed to characterize the overall pattern of immune responses. In Reference group, variability in immune responses was observed among individual animals. In contrast, HPV16 mRNA-immunized groups exhibited a predominant Th1-skewed response. These findings are consistent with the cellular immune response data, in which Th1-associated cytokines (IFN-γ, TNF-α, and IL-2) were preferentially produced in mRNA-immunized groups. Th1-dominant responses are known to support cytotoxic T cell activation, clearance of infected cells, and the establishment of long-term immunological memory, and are therefore considered a key immunological feature of vaccines targeting viral infections (42, 43). Accordingly, although neutralizing antibody titers in 1 μg HPV16 mRNA group were slightly lower than CoP, the complete protection observed against PsV challenge raises the possibility that cellular immune responses may have contributed to protection. In HPV16 mRNA 1 μg group, IFN-γ–producing T cells could not be evaluated due to a technical issue with FACS antibody, whereas TNF-α- and IL-2-producing T cells were successfully assessed. In contrast, IFN-γ–producing T cells were successfully detected in HPV16 mRNA 10 μg group. In addition, ELISpot analysis showed a tendency toward an increased frequency of IFN-γ-secreting cells compared with Reference and PBS groups. Collectively, these findings suggest that the lower-dose group elicited detectable Th1-type cellular responses, raising the possibility that immune mechanisms beyond neutralizing antibodies may have contributed to the observed protection.

HPV comprises numerous subtypes, among which HPV16 is a well-established high-risk type and a major cause of cervical cancer and other HPV-related cancers (2, 3). Accordingly, HPV16 has been widely used as a representative type for evaluating the immunogenicity and protective efficacy of HPV vaccines (1, 3, 35). Based on its clinical relevance, we selected HPV16 as the focus of this study. Accordingly, CoP proposed in this study are currently limited to HPV16. The protective efficacy of conventional VLP-based vaccines is generally restricted to target-specific HPV types, largely due to genetic variability among HPV subtypes, particularly within L1 gene region (5, 44, 45). Therefore, the immunological responses and protective effects observed in this study may not be directly applicable to other HPV types. Considering these characteristics, the development and validation of type-specific CoP for individual HPV types will be necessary. In addition, assessment of type-specific immunological profiles during multivalent vaccine design may facilitate the achievement of broader protective coverage. Future studies incorporating expanded models that include additional HPV types will be essential to validate the broader applicability of CoP and to further assess the adaptability of the vaccine platform.

Overall, this study proposes an evaluation framework for assessing the efficacy of next-generation HPV vaccines by integrating standardized immunological assays with quantitative antibody-based CoP. By applying this framework to HPV16 mRNA vaccine candidate, we demonstrated the reliability of the proposed CoP as well as the potential of mRNA-based platforms for HPV vaccine development. Although the defined evaluation criteria are currently limited to HPV16, this overall strategy can be extended to other HPV subtypes through the establishment of type-specific CoP. Furthermore, an integrated approach combining humoral and cellular immune response analyses provides a foundation for the comprehensive evaluation of immune responses. Future studies incorporating multivalent antigen designs and broader HPV type coverage will be essential to validate the generalizability of this evaluation strategy and to support the development of broadly protective HPV vaccines. Ultimately, this approach may contribute to the acceleration of HPV vaccine development by enabling rapid, quantitative, and platform-independent efficacy assessment.

## Data Availability

The original contributions presented in the study are included in the article/**Supplementary Material**. Further inquiries can be directed to the corresponding author/s.
